# Supplementary material for: Identification of photoperiod-induced specific miRNAs in the adrenal glands of Sunite sheep (Ovis aries)
Source: Front Vet Sci. 2022 Jul 22;9:888207. doi: 10.3389/fvets.2022.888207 (PMC9354845; doi:10.3389/fvets.2022.888207)
Supplement: Supplementary file 1 [file Data_Sheet_1.ZIP › Supplementary file/miRDeep2 file.zip!/miRDeep2 file/Know_Structure/LP42A1/oar-mir-380.pdf]

miRBase precursor : oar-mir-380
Total read count : 1939
oar-miR-380-5p read count : 22
oar-miR-380-3p read count : 1917
remaining reads : 0

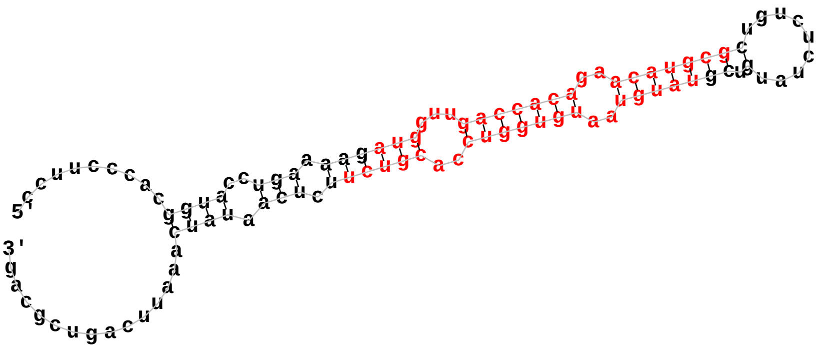

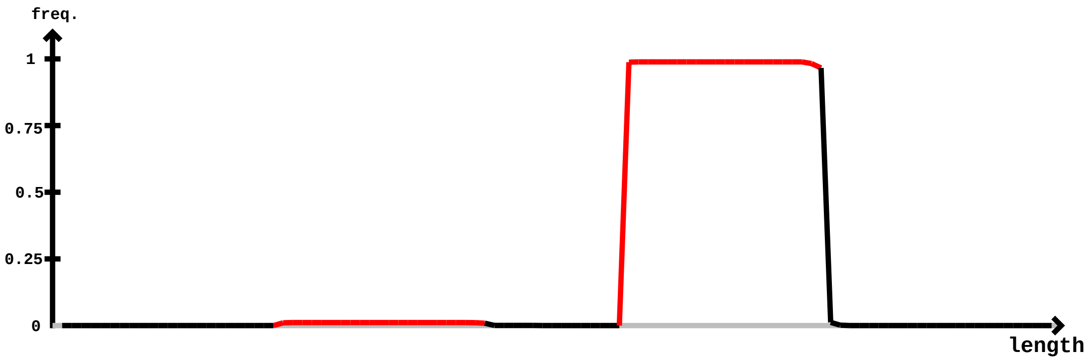


```
                                    oar-miR-380-5p
                                                                oar-miR-380-3p
5'- ccuucccacgguaccugaaaagaugguugaccacagaacaugcgcugucucuaugucguauguaaugugguccacgucuucucaauaucaaauucagucgcag  -3'   exp
    .........((((..(((.((((((...(((((((..((((((((.........).)))))))..)))))))..)))))).))).))))..............   reads   mm   sample
    ......................augguugaccacagaacaug.............................................................   1       0    seq
    ......................augguugaccacagaacaugc............................................................   4       0    seq
    ......................augguugaccacagaacaugcg...........................................................   14      0    seq
    ......................augguugaccacagaacaugcgcuguc......................................................   1       0    seq
    .......................ugguugaccacagaacaugcg...........................................................   2       0    seq
    ..........................................................uauguaaugugguccacgu..........................   12      0    seq
    ..........................................................uauguaaugugguccacguc.........................   32      0    seq
    ..........................................................uauguaaugugguccacgucu........................   1848    0    seq
    ..........................................................uauguaaugugguccacgucuu.......................   20      0    seq
    ..........................................................uauguaaugugguccacgucuuc......................   3       0    seq
    ...........................................................auguaaugugguccacgucu........................   2       0    seq
```
